# Preliminary exploratory study on differential diagnosis between benign and malignant peripheral lung tumors: based on deep learning networks

**DOI:** 10.3389/fmed.2025.1567545

**Published:** 2025-03-27

**Authors:** Yuan Wang, Yutong Zhang, Yongxin Li, Tianyu She, Meiqing He, Hailing He, Dong Zhang, Jue Jiang

**Affiliations:** ^1^Department of Ultrasound, The Second Affiliated Hospital of Xi’an Jiaotong University, Xi’an, China; ^2^Department of Ultrasound, Yaozhou District People's Hospital, Tongchuan, China; ^3^School of Automation and Intelligence, Beijing Jiaotong University, Beijing, China; ^4^Department of Ultrasound, Xi'an Electric Power Central Hospital, Xi’an, China; ^5^Department of Ultrasound, Shaanxi Provincial People's Hospital, Xi'an, China; ^6^Department of Ultrasound, Tongchuan Mining Bureau Central Hospital, Tongchuan, China; ^7^Institute of Artificial Intelligence and Robotics, Xi'an Jiaotong University, Xi’an, China

**Keywords:** artificial intelligence, ultrasound imaging, deep learning, peripheral lung tumors, differential diagnosis

## Abstract

**Background:**

Deep learning has shown considerable promise in the differential diagnosis of lung lesions. However, the majority of previous studies have focused primarily on X-ray, computed tomography (CT), and magnetic resonance imaging (MRI), with relatively few investigations exploring the predictive value of ultrasound imaging.

**Objective:**

This study aims to develop a deep learning model based on ultrasound imaging to differentiate between benign and malignant peripheral lung tumors.

**Methods:**

A retrospective analysis was conducted on a cohort of 371 patients who underwent ultrasound-guided percutaneous lung tumor procedures across two centers. The dataset was divided into a training set (*n* = 296) and a test set (*n* = 75) in an 8:2 ratio for further analysis and model evaluation. Five distinct deep learning models were developed using ResNet152, ResNet101, ResNet50, ResNet34, and ResNet18 algorithms. Receiver Operating Characteristic (ROC) curves were generated, and the Area Under the Curve (AUC) was calculated to assess the diagnostic performance of each model. DeLong’s test was employed to compare the differences between the groups.

**Results:**

Among the five models, the one based on the ResNet18 algorithm demonstrated the highest performance. It exhibited statistically significant advantages in predictive accuracy (*p* < 0.05) compared to the models based on ResNet152, ResNet101, ResNet50, and ResNet34 algorithms. Specifically, the ResNet18 model showed superior discriminatory power. Quantitative evaluation through Net Reclassification Improvement (NRI) analysis revealed that the NRI values for the ResNet18 model, when compared with ResNet152, ResNet101, ResNet50, and ResNet34, were 0.180, 0.240, 0.186, and 0.221, respectively. All corresponding *p*-values were less than 0.05 (*p* < 0.05 for each comparison), further confirming that the ResNet18 model significantly outperformed the other four models in reclassification ability. Moreover, its predictive outcomes led to marked improvements in risk stratification and classification accuracy.

**Conclusion:**

The ResNet18-based deep learning model demonstrated superior accuracy in distinguishing between benign and malignant peripheral lung tumors, providing an effective and non-invasive tool for the early detection of lung cancer.

## Introduction

Lung cancer remains one of the most prevalent and fatal cancers worldwide, with peripheral lung cancer (PLC) constituting a substantial proportion of these cases (1). PLC originates in the outer regions of the lungs and is often difficult to detect in its early stages due to subtle symptoms, leading to a high rate of misdiagnosis (2). Epidemiological studies indicate that peripheral lung tumors account for approximately 30–40% of all lung cancer diagnoses, underscoring the urgent need for effective early detection and accurate diagnosis (1, 3). Current clinical practices rely on low-dose computed tomography (LDCT) as the gold standard for lung cancer screening (4). However, while LDCT is highly effective, it involves the use of ionizing radiation, making it unsuitable for long-term monitoring, especially in individuals at high risk for lung cancer (5). Tissue biopsy, although definitive, is invasive and associated with potential complications, including bleeding and infection (2, 6). As a result, there is a growing interest in alternative, non-invasive diagnostic methods.

Ultrasound imaging has emerged as a promising non-invasive, radiation-free diagnostic tool for peripheral lung tumors, offering the advantage of high repeatability. This makes it particularly useful for monitoring patients over time and distinguishing between benign and malignant tumors (2, 5, 7). The application of ultrasound in lung tumor diagnosis has seen significant advancements in recent years. Recent studies have highlighted the improved accuracy of ultrasound techniques with the incorporation of elastography, which assesses tissue stiffness and provides valuable insights into tumor characterization (8). Additionally, the use of contrast-enhanced ultrasound (CEUS) has allowed for enhanced visualization of blood flow within tumors, further improving the ability to differentiate malignant from benign lesions (9). These innovations have made ultrasound a more reliable option for lung tumor diagnosis, particularly in settings where access to advanced imaging technologies such as CT or MRI may be limited.

However, despite these advancements, the accuracy of ultrasound diagnosis remains subject to operator-dependent variability, including factors such as experience, skill, and visual fatigue, which can lead to misjudgments (2, 5). To address these challenges, artificial intelligence (AI) techniques, particularly deep learning (DL) models, have been integrated into ultrasound imaging to improve diagnostic accuracy and consistency. Recent developments in AI have demonstrated substantial improvements in the automated analysis of ultrasound images, enabling more precise and reliable detection of lung tumors (7). Deep learning algorithms, especially convolutional neural networks (CNNs) like ResNet, have been shown to outperform traditional machine learning models by automatically detecting complex patterns and analyzing texture features that are often imperceptible to the human eye (7, 10). The ResNet architecture, known for its residual learning framework, helps mitigate the vanishing gradient problem and allows for the training of deeper neural networks, thus improving the robustness and accuracy of tumor detection (10, 11).

Therefore, incorporating AI into ultrasound imaging for PLC diagnosis has significantly reduced misdiagnosis rates and improved diagnostic confidence. AI-enhanced systems also provide real-time feedback, minimizing the effects of operator fatigue and variability, which are common limitations of traditional visual inspection (7). This study aims to develop and assess five deep learning models utilizing ultrasound images and clinical data of peripheral lung tumors. We hypothesize that these models will offer a highly accurate, non-invasive approach to differentiating benign from malignant tumors, thereby improving lung cancer screening and early diagnosis. The novelty of this research lies in the integration of ultrasound imaging with deep learning algorithms, addressing the limitations of conventional diagnostic methods and enhancing both diagnostic precision and clinical applicability.

## Materials and methods

### Study population

**Figure 1:**
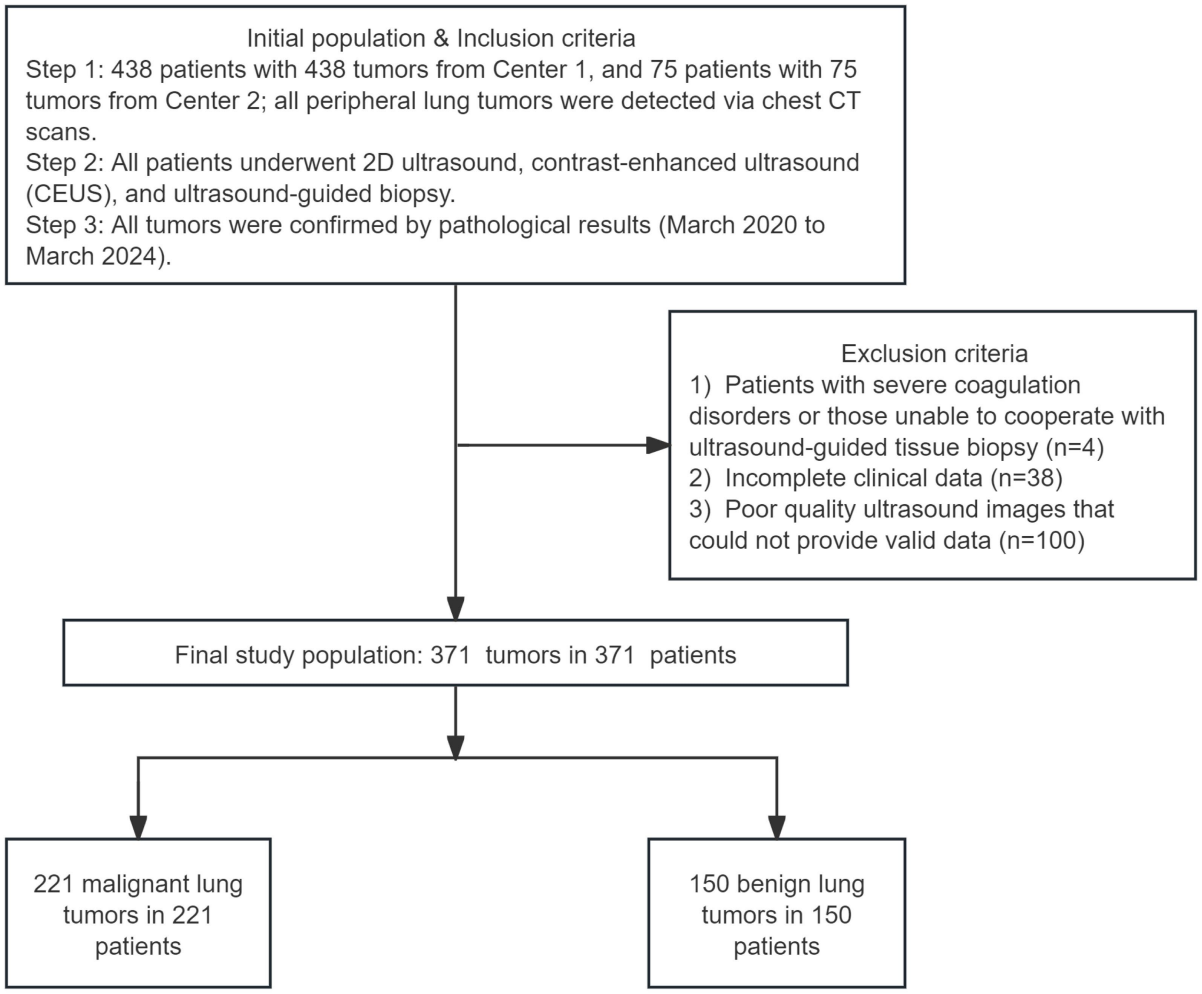
Flow diagram of the study population.

The study received approval from the institutional review board of The Second Affiliated Hospital of Xi’an Jiaotong University and Tongchuan mining bureau central hospital, which was conducted in accordance with the 1964 Declaration of Helsinki and its later amendments or comparable ethical standards. This retrospective study collected data from 513 patients with peripheral lung tumors detected via chest CT across two centers between March 2020 and March 2024. The cohort included 438 patients from Center 1 and 75 patients from Center 2, respectively. A total of 371 lung tumors, comprising 221 malignant and 150 benign cases, were included for further analysis. The inclusion criteria were as follows: (1) adult patients aged 18 years or older; (2) peripheral lung tumors detected through chest CT imaging; (3) patients who underwent ultrasound-guided tissue biopsy; (4) pathological diagnosis confirming malignant lung tumors or inflammatory lesions; (5) clear ultrasound images of adequate quality were defined by two key criteria: a minimum resolution of 1.5 millimeters and a signal-to-noise ratio (SNR) threshold of 30 dB. These standards were established to ensure sufficient image clarity and diagnostic reliability for accurate feature extraction and tumor analysis; (6) complete clinical data. The exclusion criteria included: (1) patients with severe coagulation disorders or those unable to cooperate with ultrasound-guided tissue biopsy; (2) incomplete clinical data; (3) poor quality ultrasound images that could not provide valid data (Figure 1). All patients provided informed consent, and the key contents of the informed consent form are presented in the Supplementary materials.

### Ultrasound data acquisition

Ultrasound diagnoses were performed by physicians with over 5 years of relevant experience using the Acuson Sequoia color Doppler ultrasound diagnostic system (Siemens AG, Germany), equipped with a 5C1 abdominal probe operating within a frequency range of 1.0 to 5.7 MHz. Based on lesion locations identified by CT scans, patients were positioned in supine, prone, or lateral decubitus positions to facilitate comprehensive ultrasonic examination. Clear two-dimensional ultrasound images, capturing the maximum cross-sectional area of the lesions, were retained for further analysis. To ensure consistency in data quality, all ultrasound images were acquired by trained physicians adhering to standardized imaging protocols. Images with lower resolution or insufficient signal-to-noise ratio (SNR) were excluded from the study to maintain uniformity across the dataset.

### Ultrasound image analysis and modeling

**Figure 2:**
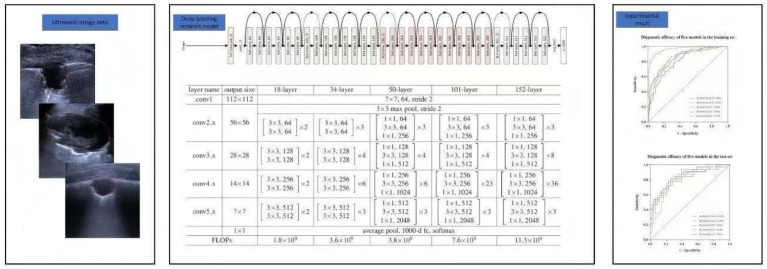
The complete experimental process.

After anonymizing patient information, the original ultrasound images were imported into the Darwin AI Research Platform for further processing. The patient information labels included the following: gender, age, biopsy site, lesion size, history of lung diseases, smoking history, and lung tumor markers. Lesion-related labels encompassed pathological results (benign or malignant), shape (round, quasi-round, triangular, wedge-shaped, or irregular), echogenicity (homogeneous or heterogeneous), presence of necrosis (present or absent), air bronchogram (present or absent), and boundary clarity (clear or unclear). Regions of interest (ROIs) were manually delineated by physicians with more than 5 years of relevant experience. In cases of disagreement, senior physicians were consulted for a definitive diagnosis. The dataset, comprising 371 patients with lung tumors, was randomly divided into a training set (*n* = 296) and a test set (*n* = 75) in an 8:2 ratio. Peripheral lung tumor ROI images and corresponding clinical data were input into the system, and the output indicated whether the tumors were benign or malignant. Based on these ultrasound imaging data and annotations, deep learning models were developed to predict the benign or malignant nature of peripheral lung tumors using five distinct algorithms: ResNet152, ResNet101, ResNet50, ResNet34, and ResNet18. Receiver operating characteristic (ROC) curves were plotted, and the area under the curve (AUC) was calculated to assess the diagnostic performance of each model. The complete experimental process is illustrated in Figure 2. During model training, we optimized hyperparameters including a learning rate of 0.001, a batch size of 64, and the Adam optimizer. We used a cosine annealing scheduler with a warm-up period for learning rate variation, and employed cross-entropy loss to guide the model in minimizing prediction errors. We implemented this study on a computer equipped with an Nvidia RTX A2000 GPU and an Intel Xeon Silver 4,208 CPU using the Darwin AI Research Platform. The average inference time per sample is approximately 50 milliseconds, measured on the aforementioned hardware. This time may vary depending on the system configuration and the complexity of the input data.

### Observation indicators

The sensitivity, specificity, accuracy, positive predictive value (PPV), and negative predictive value (NPV) of the five models in diagnosing the benignity or malignancy of peripheral lung tumors were assessed. ROC curves were plotted for each model, and the AUC was calculated to measure their diagnostic performance.

### Statistical methods

SPSS version 27.0 statistical analysis software was used to evaluate the significance of each model. Categorical data were expressed as frequencies and percentages. The classification performance of the models was assessed using the AUC, accuracy, sensitivity, specificity, PPV, and NPV derived from the ROC curves. The DeLong test was employed to compare the AUCs among the five versions of ResNet. A *p*-value of less than 0.05 was considered statistically significant, indicating a meaningful difference in performance.

## Results

### Pathological results

The study included a cohort of 371 patients diagnosed with peripheral lung tumors. Pathological analysis, based on biopsy or surgical resection, identified 221 malignant and 150 benign tumors. Detailed histological classifications are provided in Table 1. Among the malignant tumors, adenocarcinoma was the most common (26.95%), followed by squamous cell carcinoma (20.75%) and small cell carcinoma (5.12%). Benign lesions were predominantly chronic inflammation of lung tissue (26.42%) and organizing pneumonia (5.39%).

**Table 1:** Pathological results of 371 peripheral lung tumors.

Pathological findings	Benign n (%)	Malignant n (%)
Small cell carcinoma		19 (5.12)
Squamous cell carcinoma		77 (20.75)
Adenocarcinoma		100 (26.95)
Adenosquamous carcinoma		6 (1.62)
Large cell carcinoma		6 (1.62)
Malignant pleomorphic tumor		3 (0.81)
Mesenchymal sarcoma		2 (0.54)
Choriocarcinoma		2 (0.54)
Alveolar carcinoma		1 (0.27)
Metastatic renal clear cell carcinoma		2 (0.54)
Carcinosarcoma		2 (0.54)
MALT-L		1 (0.27)
Tuberculosis	11 (2.96)	
Organizing pneumonia	20 (5.39)	
Granulomatous inflammation	17 (4.58)	
Vasculitic lung injury	1 (0.27)	
Bacterial pneumonia	1 (0.27)	
Chronic inflammation of lung tissue	98 (26.42)	
Atypical adenomatous hyperplasia	2 (0.54)	

MALT-L, Mucosa-Associated Lymphoid Tissue Lymphoma.

### Performance of the deep learning models

**Figure 3:**
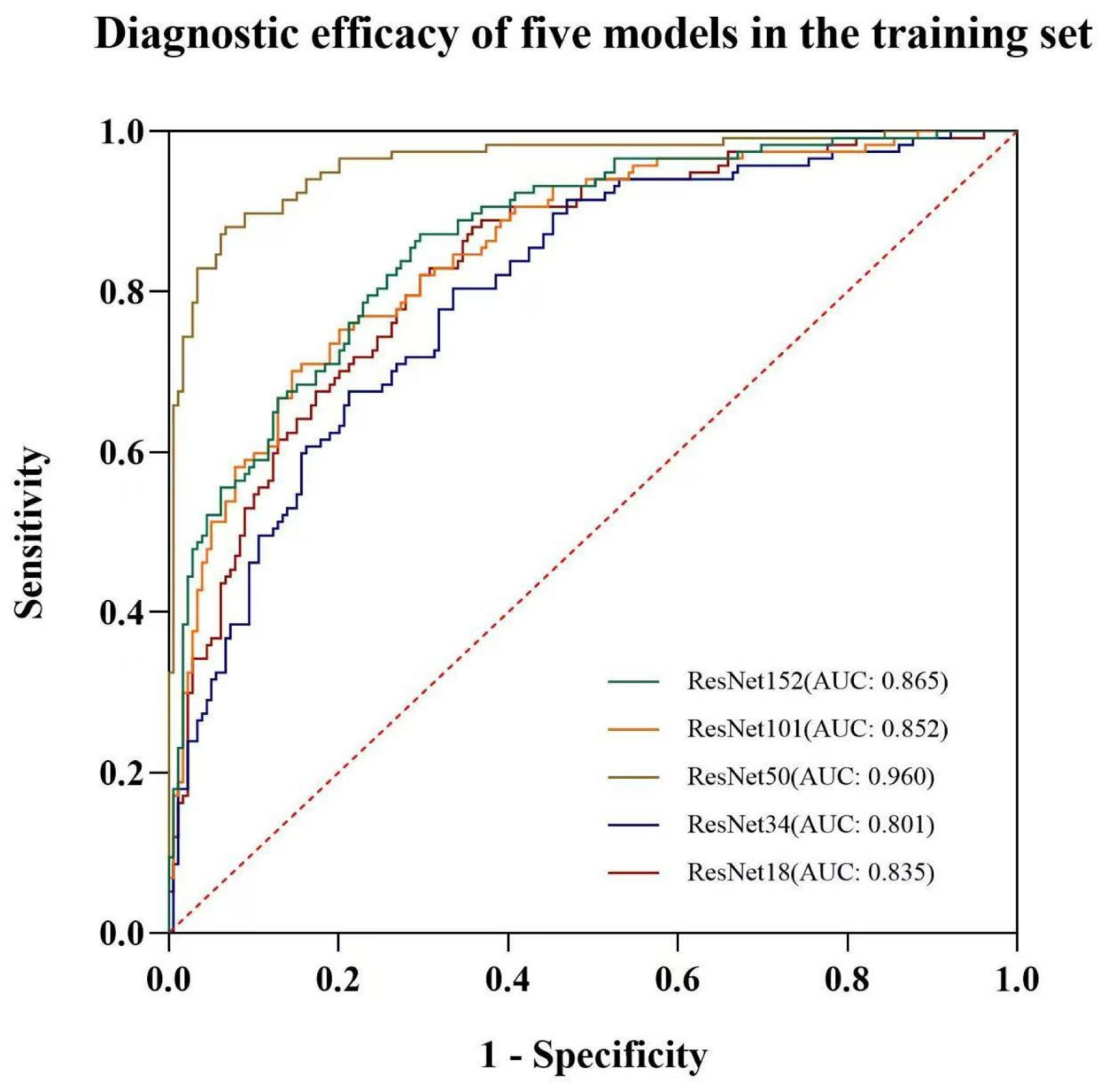
ROC curves of each deep learning model in the training set.

**Figure 4:**
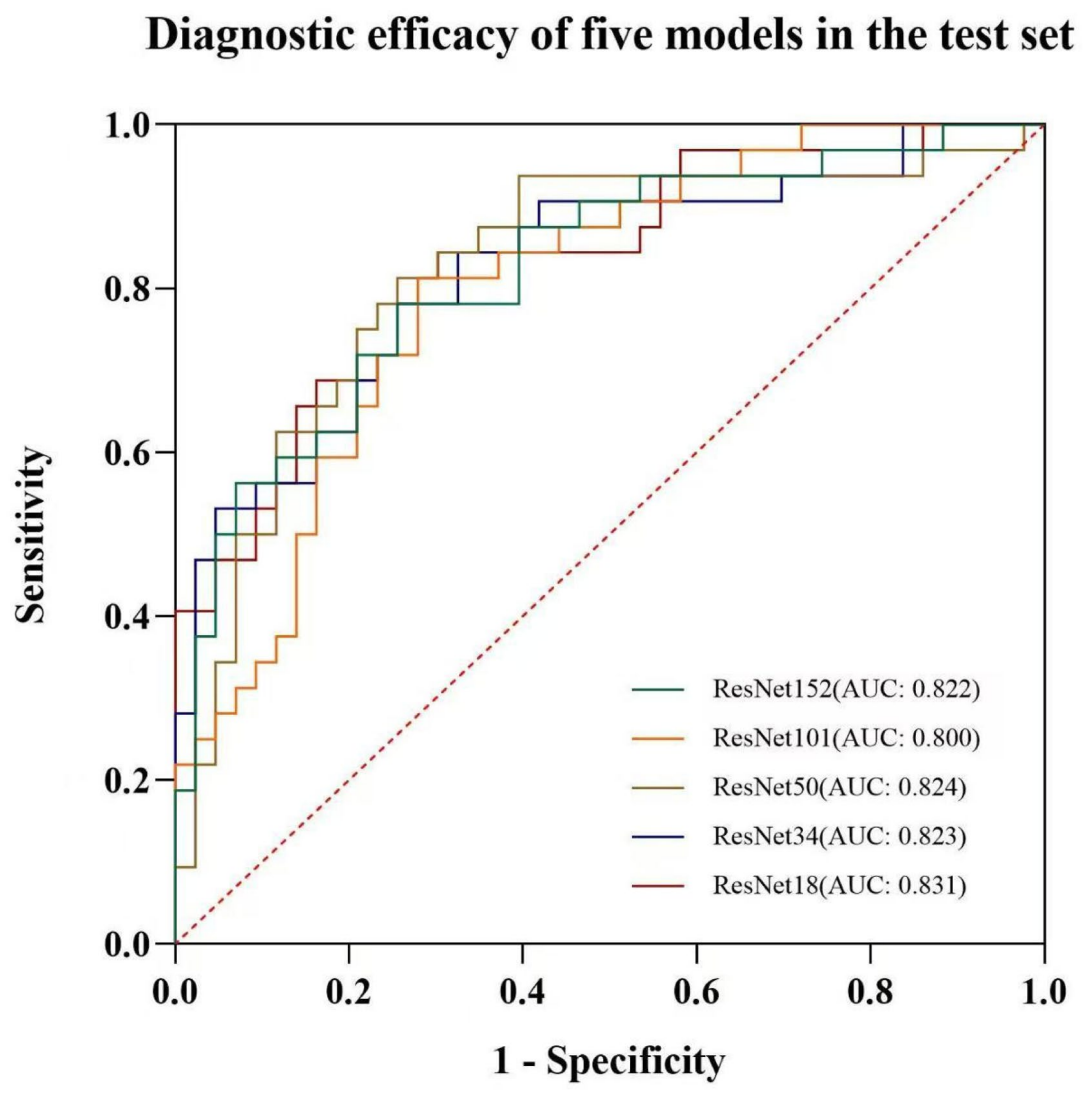
ROC plots of each deep learning model in the test set.

In the training set, the sensitivity, specificity, and diagnostic accuracy for diagnosing the benignity or malignancy of peripheral lung tumors were as follows: 87.2, 70.4, and 77.0% for Model 152; 70.1, 85.5, and 79.4% for Model 101; 88.0, 93.3, and 91.2% for Model 50; 80.3, 66.5, and 72.0% for Model 34; and 82.1, 70.4, and 75.0% for Model 18. In the test set, the corresponding values were 78.1, 74.4, and 76.0% for Model 152; 81.3, 72.1, and 76.0% for Model 101; 81.3, 74.4, and 77.2% for Model 50; 78.1, 74.4, and 76.0% for Model 34; and 84.4, 69.8, and 76.0% for Model 18 (Table 2). The areas under the receiver operating characteristic (ROC) curves (AUCs) for the five models in the training set were 0.865, 0.852, 0.960, 0.803, and 0.835, respectively (Figure 3). In the test set, the AUCs were 0.822, 0.800, 0.824, 0.823, and 0.831, respectively (Figure 4).

**Table 2:** Comparison of the performance of each deep learning model in the training and test sets.

	ResNet152		ResNet101		ResNet50		ResNet34		ResNet18	
	Training set	Test set	Training set	Test set	Training set	Test set	Training set	Test set	Training set	Test set
AUC	0.865	0.822	0.852	0.800	0.960	0.824	0.803	0.823	0.835	0.831
ACC	0.770	0.760	0.794	0.760	0.912	0.772	0.720	0.760	0.750	0.760
SEN	0.872	0.781	0.701	0.813	0.880	0.813	0.803	0.781	0.821	0.844
SPE	0.704	0.744	0.855	0.721	0.933	0.744	0.665	0.744	0.704	0.698
F1score	0.750	0.735	0.729	0.743	0.888	0.754	0.694	0.735	0.722	0.750
PPV	0.658	0.694	0.759	0.684	0.896	0.703	0.610	0.694	0.644	0.675
NPV	0.894	0.821	0.814	0.838	0.923	0.842	0.838	0.821	0.857	0.857
*p* value	2.648E-26	2.000E-06	1.237E-24	1.000E-05	4.249E-39	2.000E-06	2.2448E-18	2.000E-06	2.089E-22	1.000E-06
95%CI	0.823,0.906	0.725,0.919	0.808,0.896	0.701,0.899	0.937,0.983	0.724,0.924	0.750,0.851	0.726,0.921	0.788,0.881	0.738,0.925

AUC, Area Under the Curve; ACC, Accuracy; SEN, Sensitivity; SPE, Specificity; F1score, F1 Score; PPV, Positive Predictive Value; NPV, Negative Predictive Value; 95%CI, 95% Confidence Interval.

DeLong’s test revealed statistically significant differences in the AUCs between the five models, with ResNet18 outperforming the other models in terms of predictive accuracy and discriminatory power. NRI analysis revealed substantial improvements in the ResNet18 model compared to the other models. The NRI values for each model were as follows: ResNet152 (NRI = 0.180), ResNet101 (NRI = 0.240), ResNet50 (NRI = 0.186), and ResNet34 (NRI = 0.221). All NRI comparisons yielded statistically significant results, with *p*-values less than 0.05. These findings further substantiate that the ResNet18 model outperformed the other models in terms of reclassification ability and predictive accuracy.

## Discussion

The current study successfully developed a deep learning model based on ultrasound imaging to differentiate benign and malignant peripheral lung tumors. This model, utilizing the ResNet18 architecture, demonstrated superior performance with an AUC of 0.835 in the training cohort and 0.831 in the testing cohort, compared to models based on other ResNet architectures (ResNet152, ResNet101, ResNet50, and ResNet34). The ResNet18 model significantly outperformed the other models in terms of predictive accuracy, discriminatory power, and reclassification ability, making it a promising tool for early lung cancer detection.

In recent years, deep learning techniques have significantly improved lung tumor diagnosis across imaging modalities like CT, PET/CT, and ultrasound. CT and PET/CT are commonly used in clinical settings for their high spatial resolution and detailed anatomical information. Studies have shown that deep learning can enhance the performance of these techniques in detecting malignancies. For example, Yang et al. (12) used deep convolutional neural networks (CNNs) to analyze CT scans, achieving over 90% accuracy in distinguishing between benign and malignant pulmonary nodules (12).

Despite their high diagnostic accuracy, CT and PET/CT have several limitations. Both involve ionizing radiation, which can lead to cumulative exposure risks, especially with repeated imaging. Additionally, PET/CT scanners are expensive and less accessible, limiting their use in some clinical settings (13). In contrast, ultrasound offers significant advantages, especially when combined with deep learning techniques. Unlike CT and PET/CT, ultrasound does not involve ionizing radiation, making it a safer option for patients, particularly in long-term monitoring. Ultrasound is also more cost-effective, portable, and accessible, making it ideal for resource-limited settings. Recent studies, such as Liu et al. (14), have shown that deep learning applied to ultrasound images can achieve 88% sensitivity and 85% specificity for early lung cancer detection (14). Ultrasound’s real-time imaging capability provides immediate feedback, aiding quick decision-making, and it can be performed at the patient’s bedside, making it a valuable tool for point-of-care diagnosis (15). However, ultrasound does have limitations. Its quality is highly dependent on the skill of the operator, which can lead to inconsistent results. Ultrasound may also struggle to visualize deeper lung tissues due to interference from air in the lungs and difficulty distinguishing solid tumors from surrounding structures. Additionally, deep learning algorithms for ultrasound are still underdeveloped compared to those for CT and PET/CT, which have more standardized images (16). Despite these challenges, ultrasound’s non-invasive nature, lack of ionizing radiation, portability, and real-time feedback make it a promising tool for lung cancer detection, particularly when enhanced by advanced deep learning techniques (17–20).

The reasons for choosing ResNet to construct the predictive model in this study, rather than other architectures (e.g., EfficientNet, Vision Transformer), are as follows: firstly, ResNet introduces the concept of residual connections, which address the vanishing gradient problem by allowing gradients to flow more easily through deeper layers (21). This enables the training of much deeper networks without the degradation in performance typically seen in conventional deep networks (22). In comparison, while EfficientNet and ViT also achieve high performance, they do not inherently mitigate the vanishing gradient problem to the same extent, especially in very deep architectures (23, 24). Secondly, ResNet excels in feature extraction, leveraging its deep architecture and residual blocks, which enables it to capture more complex patterns and fine-grained details in images (25). This is particularly useful in medical imaging, where subtle differences in image features are crucial for accurate diagnosis (26). EfficientNet and ViT, while powerful, may not always achieve the same level of fine-grained feature extraction, particularly for highly specialized tasks such as detecting peripheral lung tumors (27, 28). Thirdly, ResNet offers a good balance between model depth and computational cost (29). Although deeper networks typically require more computation, the residual connections in ResNet allow for more efficient training and inference compared to other architectures like ViT, which can be computationally expensive due to the self-attention mechanism (30). EfficientNet, on the other hand, optimizes the trade-off between accuracy and efficiency, but its scaling strategy might still be less computationally efficient than ResNet in certain applications (31, 32). Fourthly, ResNet has been extensively validated across a wide range of medical imaging tasks, demonstrating robustness and reliability (33). It has a proven track record in both image classification and segmentation tasks (34, 35). While architectures like EfficientNet and ViT also show great promise, ResNet’s long-standing success in medical imaging, along with its established frameworks for fine-tuning, makes it a reliable choice for clinical applications (36). In summary, ResNet’s advantages lie in its deep network capability, residual learning to avoid gradient issues, efficient feature extraction, and computational practicality, all of which make it particularly suitable for medical image analysis compared to other architectures like EfficientNet and Vision Transformer (37).

This study evaluated the performance of various ResNet architectures in predicting the benign or malignant nature of peripheral lung tumors. The findings revealed that the ResNet18-based model outperformed those based on ResNet152, ResNet101, ResNet50, and ResNet34. A deeper analysis, considering both the algorithmic network architecture and the dataset, provides valuable insights into the factors contributing to this result. Firstly, ResNet18, being a relatively shallow model, has fewer layers compared to deeper networks like ResNet152. This means it requires less computational power, leading to faster training times and quicker inference speeds. This can be important in resource-constrained environments, such as embedded systems or mobile devices (38). Secondly, with fewer parameters and layers, ResNet18 demands less memory for storage and computation. This is beneficial in settings where memory is limited, and it can be crucial for deployment in edge devices or situations where there is a need to optimize for power consumption and storage (39). Thirdly, in many real-world tasks, particularly when the dataset size is not large enough to fully leverage the capacity of deeper networks, a smaller model like ResNet18 can avoid overfitting. Larger models like ResNet152, due to their increased number of parameters, may overfit on smaller datasets if not properly regularized (40). Fourthly, when performing transfer learning, using a smaller model like ResNet18 may lead to easier fine-tuning, especially on datasets where the target task is relatively simpler. The smaller number of parameters also means it is easier to modify the model for specific use cases without requiring excessive computational resources (41). Fifthly, while deeper models (like ResNet152) might provide better performance on very large datasets, ResNet18 offers a good balance between performance and computational efficiency. It can achieve decent accuracy with much less computational cost, making it ideal for practical applications where speed is essential (42). Sixthly, due to fewer layers and parameters, ResNet18 is more interpretable and simpler to analyze compared to deeper architectures. This simplicity can be beneficial for debugging or understanding how the network is making decisions (43). Overall, ResNet18 is preferred in situations where computational efficiency, memory constraints, or training time are critical considerations, while deeper ResNets like ResNet152 might be more suitable for large-scale datasets and applications that demand the highest performance.

There were still several limitations in this study. Firstly, while the results achieved are promising, the sample size remains limited. Future studies should include a larger cohort of patients, and external validation using independent test datasets is essential to confirm the generalizability of the model. Secondly, all data in this study were sourced from two centers. Therefore, additional multi-center research is required to enhance the robustness and applicability of the findings in broader clinical settings. Thirdly, the model is employed solely for the classification of benign and malignant lung tumors. A more comprehensive analysis focusing on their pathological classification will be conducted in subsequent studies.

## Conclusion

The deep learning model based on ResNet18 demonstrated superior performance in differentiating between benign and malignant peripheral lung tumors compared to other ResNet-based models. The ResNet18 model exhibited statistically significant improvements in predictive accuracy and discriminatory power, as evidenced by ROC analysis and NRI evaluations. These findings highlight the potential of ultrasound imaging, in combination with advanced deep learning techniques, as an effective and non-invasive approach for the early detection of lung cancer. This study supports the clinical application of ResNet18 in enhancing diagnostic accuracy and risk stratification for lung lesions, contributing to more timely and accurate diagnosis of lung cancer.

## Data Availability

The datasets presented in this article are not readily available. Requests to access the datasets should be directed to Yuan Wang, wy18329591877@163.com.
