# Supplementary material for: Preliminary exploratory study on differential diagnosis between benign and malignant peripheral lung tumors: based on deep learning networks
Source: Front Med (Lausanne). 2025 Mar 27;12:1567545. doi: 10.3389/fmed.2025.1567545 (PMC11983456; doi:10.3389/fmed.2025.1567545)
Supplement: Supplementary file 1 [file Table_1.docx]

Supplementary Material

# Supplementary Data：the basic content of the informed consent form

**The Second Affiliated Hospital of Xi’an Jiaotong University**

**Informed Consent for Ultrasound-guided Puncture Diagnosis and Treatment**

| Name |  | Sex |  | Age |  | Department |  | Outpatient/Inpatient number |  |
| --- | --- | --- | --- | --- | --- | --- | --- | --- | --- |
| Preliminary diagnosis: | | | | | | | | | |
| Name of interventional treatment: ultrasound-guided percutaneous biopsy  Anesthesia method: local anesthesia  The operation, a minimally invasive exam, aims for quick histological diagnosis. While common and mature, medical variables and individual differences may cause adverse reactions, potentially leading to organ dysfunction and life-threatening situations.  The main adverse reactions are:  1. Anesthesia allergy, causing circulatory and respiratory problems.  2. Infections.  3. Bleeding at the operation site and damage to nearby organs.  4. Local pain and hemoptysis after the procedure.  5. Failure to achieve expected results and unclear pathological diagnosis due to material issues.  6. Scale pressure and short-term shock from stress, hypoglycemia.  7. Other unforeseen life-threatening or disabling incidents.  Although the occurrence of such situations is rare and most symptoms are mild, with quick recovery after treatment, they cannot be completely avoided with current medical technology. In case of serious complications, emergency observation or surgery may be necessary.  We respect patients' and their families' rights to know and choose. Please read carefully and sign with your comments.  The attending physician:  Date:  Have hypertension, coronary heart disease history hit "√" hypertension: Yes □ No □  Coronary heart disease: Yes □ No □  Whether to take anticoagulants, in line with the dozen "√" (aspirin, warfarin, etc.): Yes □ No □  I've read and grasped the above. I understand the exam's necessity and potential risks. I consent to the exam and will bear consequences. I won't refuse payment or discharge if issues arise.  Patient's/Family's Sign: ____ and patient relationship: ___ contact number: _____ Date: _____ | | | | | | | | | |

**Central Hospital of Tongchuan Mining Bureau**

**Informed Consent for Ultrasound-guided Puncture Diagnosis and Treatment**

| Name |  | Sex |  | Age |  | Department |  | Outpatient/Inpatient number |  |
| --- | --- | --- | --- | --- | --- | --- | --- | --- | --- |
| Preliminary diagnosis: | | | | | | | | | |
| Name of interventional treatment: ultrasound-guided percutaneous biopsy  Anesthesia method: local anesthesia  The operation, a minimally invasive exam, aims for quick histological diagnosis. While common and mature, medical variables and individual differences may cause adverse reactions, potentially leading to organ dysfunction and life-threatening situations.  The main adverse reactions are:  1. Anesthesia allergy, causing circulatory and respiratory problems.  2. Infections.  3. Bleeding at the operation site and damage to nearby organs.  4. Local pain and hemoptysis after the procedure.  5. Failure to achieve expected results and unclear pathological diagnosis due to material issues.  6. Scale pressure and short-term shock from stress, hypoglycemia.  7. Other unforeseen life-threatening or disabling incidents.  Although the occurrence of such situations is rare and most symptoms are mild, with quick recovery after treatment, they cannot be completely avoided with current medical technology. In case of serious complications, emergency observation or surgery may be necessary.  We respect patients' and their families' rights to know and choose. Please read carefully and sign with your comments.  The attending physician:  Date:  Have hypertension, coronary heart disease history hit "√" hypertension: Yes □ No □  Coronary heart disease: Yes □ No □  Whether to take anticoagulants, in line with the dozen "√" (aspirin, warfarin, etc.): Yes □ No □  I've read and grasped the above. I understand the exam's necessity and potential risks. I consent to the exam and will bear consequences. I won't refuse payment or discharge if issues arise.  Patient's/Family's Sign: ____ and patient relationship: ____ contact number: _____Date: ____ | | | | | | | | | |
